# Early-Stage Vision and Perceptual Imagery in Autism Spectrum Conditions

**DOI:** 10.3389/fnhum.2019.00337

**Published:** 2019-10-01

**Authors:** Rebeka Maróthi, Katalin Csigó, Szabolcs Kéri

**Affiliations:** ^1^Nyírö Gyula National Institute of Psychiatry and Addictions, Budapest, Hungary; ^2^Department of Cognitive Science, Budapest University of Technology and Economics, Budapest, Hungary; ^3^Department of Physiology, University of Szeged, Szeged, Hungary

**Keywords:** autism, perception, mental imagery, early vision, lateral masking

## Abstract

Autism spectrum conditions (ASC) are characterized by multifaceted alterations in visual perception and mental imagery. However, the interaction between early-stage visual perception and imagery has not been explored. We recruited 40 individuals with ASC and 20 neurotypical control volunteers to participate in a lateral masking task. Participants detected a luminance-contrast target pattern (Gabor patch) flanked by two collinear masks. The flanking masks inhibit target detection at small target-mask distances and facilitate target detection at intermediate target-mask distances. In the perceptual task, the masks appeared adjacent to the target. In the imagery task, participants imagined the masks immediately after seeing them. Results revealed that individuals with ASC characterized by exceptional visuoconstructional abilities (enhanced Block Design performance; *n* = 20) showed weaker inhibition at small target-mask distances and stronger facilitation at intermediate target-mask distances relative to the controls. Visual imagery was markedly dampened in ASC regardless of the visuoconstructional abilities. At the behavioral level, these results indicate increased facilitation *via* lateral connections in the primary visual cortex (V1) of individuals with ASC who exhibit exceptional visuoconstructional abilities, together with less efficient mental imagery.

## Introduction

Autism spectrum conditions (ASC) are characterized by atypical neurodevelopmental patterns, often leading to impairments in social interactions, communication, and inflexible behavior. Additionally, perceptual anomalies are an important aspect of ASC (Dakin and Frith, 2005; Simmons et al., 2009; Mottron, 2011; Robertson and Baron-Cohen, 2017), as outlined by the weak central coherence framework (Happé and Frith, 2006) and the enhanced perceptual functioning hypothesis (Mottron et al., 2006). The core feature of these theories is the abnormal interaction between bottom-up and top-down processes (Pellicano and Burr, 2012).

During low-level (early-stage) visual perception, the sensory system automatically extracts elementary information from external objects (e.g., lightness, color, stereopsis, and motion). Low-level vision is bottom-up and data-driven because it originates with the stimulation of rods and cones in the retina, and this information is immediately forwarded to the visual cortex *via* the lateral geniculate nucleus. In contrast, top-down processes refer to the effect of previous knowledge and mental effort on perception and recognition. In other words, the brain makes predictions and inferences based on past experiences and memories (Pellicano and Burr, 2012). A typical example is mental imagery (visualizing or “seeing in the mind’s eye”), which is similar to perceptual experience, but occurs in the absence of external objects: the individual retrieves images from the memory and intentionally maintains this information in the focus of consciousness (Kosslyn et al., 2006).

A paradigm shift in the research of sensory cortical areas led to the recognition that the primary visual cortex (V1) is not a passive “blackboard” for the bottom-up perception of basic object features. It turned out that top-down signals from higher-level cortical areas (e.g., prefrontal cortex and anterior cingulate cortex) modulate neural activity even in the V1 during working memory, retrieval, and mental imagery (Pearson et al., 2015; Roelfsema and de Lange, 2016). Caron et al. (2006) showed enhanced performance in the detection of simple visual stimuli and superior discrimination of first-order gratings in ASC, which indicates a heightened functioning of V1. However, despite extensive research efforts, we behold scarce knowledge about the interaction between bottom-up and top-down processes in the earliest stage of visual information processing in ASC.

**Figure 1:**
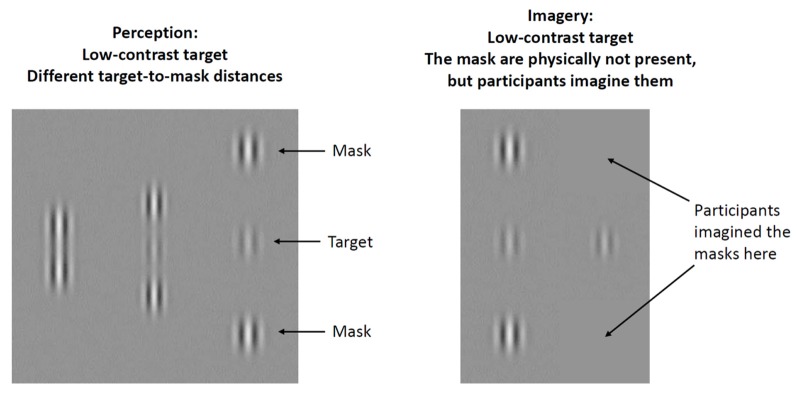
Illustration of experimental stimuli. A low-contrast, central Gabor patch (target) was flanked by two lateral masks at three different target-mask distances (1λ, 3λ, and 6λ). Contrast threshold was measured for the target. At short target-mask distances (1λ), masks inhibit target detection (higher contrast threshold is needed for detection), at intermediate target-mask distances (3λ) masks facilitate target detection (lower contrast threshold is needed for detection), whereas at long target-mask distances (6λ) masks have a negligible effect.

The lateral masking task provides a unique opportunity for the behavioral assessment of bottom-up perception and top-down imagery at the level of the V1 (Ishai and Sagi, 1995; Kéri, 2015; Maróthi and Kéri, 2018; Figure 1). In the perceptual part of the task, participants detect a central low-contrast target (Gabor patch) vertically flanked by two high-contrast masks. Gabor patches are small, bean-shaped objects consisting of alternating, brighter and darker regions, providing an optimal stimulus for the V1 (Ishai and Sagi, 1997; Figure 1). The luminance contrast of the target Gabor patch is defined by the difference in its brightness and that of the background. The flanking masks facilitate the detection of the central target if they fall within an intermediate distance from the target: participants are able to detect a target with low contrast. However, the masks have an inhibitory effect if the target-mask distance is small, that is, a high contrast is necessary for target detection. It has been postulated that the effect of masks can be attributed to lateral interactions between neuronal groups and their feedback modulation in the V1. These lateral (horizontal) interactions are thought to be mediated by short-range connections between neurons responding to similar visual stimuli (Polat and Sagi, 1993; Polat et al., 1998; Angelucci et al., 2002; Crook et al., 2002). Interestingly, we can observe similar masking effects when participants only imagine the previously presented masks, which indicates an interplay between bottom-up and top-down processes (Ishai and Sagi, 1995). At the neural level, cortical cells responding to simple visual stimuli and their lateral interactions might be activated by signals from higher-level extrastriate and prefrontal areas in the absence of external stimuli (Freeman et al., 2003). Moreover, the imagery-induced facilitation of target detection persists for several minutes after viewing the masks, which may point to the existence of a monocular, orientation-specific, low-level iconic memory system that stores the sensory trace of the masks and enables the reactivation of these low-level representations during top-down mental imagery (Ishai and Sagi, 1995). It has also been shown that the top-down imagery effect is weaker than the facilitation induced by the physical presence of the masks and is especially pronounced at intermediate target-mask distances (Ishai and Sagi, 1997). It must be underlined that higher-level top-down control during imagery is different from early-stage feedback mechanisms related to short-term memory and to the modulation of target-mask interactions (Gilbert et al., 2000; Angelucci et al., 2002; Freeman et al., 2003; Summerfield and Egner, 2009). Anatomical and physiological measurements from the macaque mapped the role of intra-areal V1 lateral connections and inter-areal feedback connections to V1 in spatial summation. Monosynaptic lateral connections within V1 mediated interactions in the spatial summation field of neurons, but feedback circuits from extrastriate cortex to V1 were needed for the full spatial range of center-surround interactions necessary for the contextual modulation and global-to-local integration of visual signals (Angelucci et al., 2002).

Kéïta et al. (2011) applied the lateral masking task to assess the functional integrity of lateral interactions in the visual cortex of individuals with ASC. The authors measured contrast thresholds for a centrally presented target Gabor patch flanked by collinear masks at different distances. As expected, both ASC and control groups showed heightened target sensitivity when the target-mask distance was intermediate. Strikingly, this facilitation was significantly greater in the ASC group relative to the control group (Kéïta et al., 2011). The authors concluded that atypical visual functions in ASC can be explained by altered lateral interactions in the visual cortex responsible for the earliest stage of feature extraction (e.g., luminance, hue, spatial frequency, and orientation of objects). However, other groups failed to replicate these findings (Jachim et al., 2015; Dickinson et al., 2018), possibly because of substantial differences in samples and psychophysical methods. There are no studies aimed to evaluate imagery in the same experimental setting.

To resolve these controversial results, it is critical to take into consideration that individuals with ASC exhibit a high degree of variation in the development of visuospatial functions (Muth et al., 2014), and it may be related to mental imagery (Soulières et al., 2011). Some of them display outstanding performances (phenotypic peaks) on tasks assessing visuoconstructional abilities (e.g., Block Design and Raven’s Progressive Matrices) relative to other cognitive functions (Caron et al., 2006). The Block Design test is a part of non-verbal IQ assessment, during which participants are asked to rearrange blocks with different color patterns on their sides to match a predefined template (Wechsler, 1997). Regarding mental imagery, individuals with ASC who exhibit a Block Design peak performed better on a mental rotation task than non-autistic controls and ASC individuals with no Block Design peak. This indicates their heightened imagery ability to form, access, and manipulate mental images (Soulières et al., 2011), which is in line with the classic idea that some people with ASC think in pictures (Grandin, 1995; Kana et al., 2006; Heaton et al., 2008; Soulières et al., 2009; Sahyoun et al., 2010). However, it is not known whether enhanced visuoconstructional and imagery abilities are related to the earliest level of visual information processing, and how top-down factors interact with bottom-up perception.

Therefore, we tested the following hypotheses: (1) individuals with ASC exhibiting a Block Design peak (ASC Peak) show higher facilitation in the lateral masking task relative to neurotypical controls and ASC with no such phenotypic peak (ASC Non-Peak); and (2) based on the results of Soulières et al. (2011), we hypothesized that individuals with ASC Peak show enhanced mental imagery abilities.

## Materials and Methods

### Participants

We recruited 40 individuals living with ASC and 20 neurotypical control subjects matched for sex, age, and education by contacting self-help and community support groups (Table 1). Individuals with Asperger’s syndrome were not included. The study was conducted at the National Institute of Psychiatry and Addictions, Budapest, Hungary. For the diagnosis, we used the Autism Diagnostic Interview-Revised (ADI-R; Lord et al., 1994) and the Autism Diagnosis Observation Schedule (ADOS-G, module 3 or 4; Lord et al., 2000). The interviews were conducted by trained clinical psychologists who were blind to the aim of the study. Individuals with neurological and psychiatric disorders other than ASC did not participate in the study. None of our volunteers received psychotropic medications. There were four sharp perceivers in the control group (visual acuity better than 20/20), and seven in the ASC group (Sloan visual acuity chart, Precision Vision, LaSalle, IL, USA; Tavassoli et al., 2011). The remaining participants had normal (20/20) visual acuity. There was no evidence of strabismus in our sample as confirmed by Hirschberg corneal reflex test.

**Table 1:** Participants of the study.

	Autism spectrum disorder		Neurotypical controls	*F*	*p*
	Visuoconstructional Peak	Non-Peak			
Male/female	20/0	20/0	20/0	-	-
Age (years)	23.6 (6.4)	24.0 (5.9)	23.9 (7.0)	0.02	0.98
Education (years)	12.5 (4.3)	12.9 (3.6)	13.0 (5.1)	0.07	0.93
Full-scale IQ	104.2 (11.9)	102.0 (9.2)	101.5 (10.2)	0.38	0.69
Performance IQ*	112.1 (12.2)	101.8 (9.1)	100.5 (9.7)	7.44	0.001
Verbal IQ	96.4 (11.3)	102.1 (9.0)	102.7 (10.9)	2.22	0.12
Block Design values*	18.0 (4.3)	11.3 (3.0)	10.7 (2.4)	29.63	<0.001
Autism Diagnostic Interview-Revised					
Social	22.7 (5.3)	22.1 (6.7)	-	0.10	0.76
Communication	17.6 (4.9)	17.2 (4.2)	-	0.08	0.78
Behavior	6.4 (2.5)	5.9 (2.3)	-	0.43	0.51

Data are mean (standard deviation). The table shows between-group comparisons with one-way ANOVAs. *Peak > Non-Peak = Controls (*p* < 0.05, Tukey’s HSD tests).

All participants gave written informed consent, and the study was approved by the National Medical Research Council (Budapest, Hungary; ETT-TUKEB 18814). Based on the permission of the National Medical Research Council, the study was also approved by the local ethics board of the National Institute of Psychiatry and Addictions (Budapest, Hungary). All research was performed in accordance with relevant guidelines and regulations.

### Classification Based on Block Design Performance

We used the Block Design subtest of the Wechsler Intelligence Scale (WAIS-III; Wechsler, 1997) to determine whether participants with ASC exhibited an outstanding visuospatial performance or not. By using the criteria of Soulières et al. (2011), a significant strength or peak (less than 5% of the general population and approximately 40%–50% of individuals with ASC) in the Block Design subtest was defined as a difference of at least 3.9 points between the standard score on the Block Design subtest and the average of all standard WAIS-III scores for a given individual. From a larger sample, we selected 20 individuals with Block Design peak (ASC Peak, Block Design score range: 4.2–7.5, three sharp perceivers), and 20 individuals without such performance strength (ASC Non-Peak, Block Design score range: −0.5 to 1.1, four sharp perceivers). The Block Design classification process was blinded.

### Lateral Masking

The procedure was identical to that used in our previous studies (Kéri, 2015; Maróthi and Kéri, 2018), and here we only describe a short summary (Figure 1). We measured contrast threshold for a target Gabor patch when it was flanked by two lateral collinear masks with different target-to-mask distances (perception condition) or when the masks were not physically present, but the participant was instructed to imagine them (imagery condition). Target and mask Gabor patches appeared on a MultiSync PA301W monitor (NEC, Itasca, IL, USA; display area: 10° by 10°; viewing distance: 150 cm; mean display luminance: 50 cd/m^2^; resolution: 10-bit), characterized by spatial frequency (6.7 cycles/degree for both target and masks), luminance-contrast (masks: 40% of Michelson-contrast), and Gaussian envelope size (0.15°). The gamma function of the screen was linearized by using a lookup table. We selected the spatial frequency of the stimuli because it provided a reliable masking effect in our previous studies (Kéri, 2015; Maróthi and Kéri, 2018).

Before the measurements, participants received a practice run to ensure that the task instructions were clear and understandable (one block of 50 trials, including perception and imagery). Each trial was initiated by the subject who pressed a key on the computer keyboard. Four subsequent phases comprised a trial: blank pre-stimulus period (500 ms), first stimulus period (90 ms), blank inter-stimulus interval (1,000 ms), and second stimulus period (90 ms). We asked the participants to indicate whether the target patch was flashed during the first or the second stimulus period by pressing two different keys (“0” or “9”). We administered nine randomized blocks of 50 trials during which the target-mask distance was constant. The center-to-center target-mask distance was depicted by the wavelength of the Gabor patched (inverse of the spatial frequency, λ): 1, 3, or 6λ. Each perception block was immediately followed by a corresponding imagery block during which participants were requested to imagine the masks at the same distance as they saw in the preceding perception block. The contrast threshold for a target without masks was measured in a separate block.

We used a staircase method, converging at 79.4% performance, to measure the target contrast threshold (Levitt, 1971). After three consecutive correct responses, the contrast was decreased by 0.1 log unit, whereas after an incorrect response, the contrast was increased by 0.1 log unit. A block was terminated after eight reversals (when contrast was lowered and subsequently increased), and the final contrast threshold was the average of the last seven reversals. The changes in the contrast threshold were expressed relative to the baseline (no mask) condition. Positive values mean that contrast detection thresholds increased in the presence of the masks, whereas negative values indicate that the detection thresholds were reduced. Contrast threshold changes were characteristically affected by the target-to-mask distance (1λ: masks inhibit target detection; 3λ: masks facilitate target detection; 6λ: masks have negligible effects on target detection; Polat and Sagi, 1993).

### Fatigue and Motivation

To measure changes in mental efforts and motivation during the experiment, we used the Multidimensional Fatigue Inventory (MFI) at the beginning and at the end of the procedure. The MFI consists of 20 items defining four categories: general fatigue, mental fatigue, reduced activities, and motivation. Each item is rated on a 5-point Likert scale (from “True” to “Not true”). Lower MFI points mean greater fatigue (Smets et al., 1995; Gergelyfi et al., 2015).

### Data Analysis

We used STATISTICA 13.1 software (TIBCO, Palo Alto). First, normality of data distribution and homogeneity of variance were checked with Kolmogorov–Smirnov and Levene’s tests, respectively. The main dependent measure was contrast threshold changes in the lateral masking task, which was entered into analysis of variances (ANOVAs), followed by Tukey’s Honestly Significant Difference (HSD) tests. In the ANOVA, the between-subjects factor was the three groups (ASC Peak, ASC Non-Peak, and controls), and the within-subjects factor was the three target-mask distances. We also explored whether facilitation and inhibition were significant at different target-mask distances by comparing the contrast threshold value at each distance with the baseline (no mask) value with *t*-tests (two-tailed, Bonferroni-corrected for multiple comparisons). Demographic parameters and IQ were compared in the three groups with one-way ANOVAs. Cohen’s effect sizes were calculated for the head-to-head comparison of the groups. Pearsons’s product moment correlation coefficients were determined between contrast threshold changes and Block Design scores. The level of statistical significance was set at α < 0.05.

## Results

### Perception

**Figure 2:**
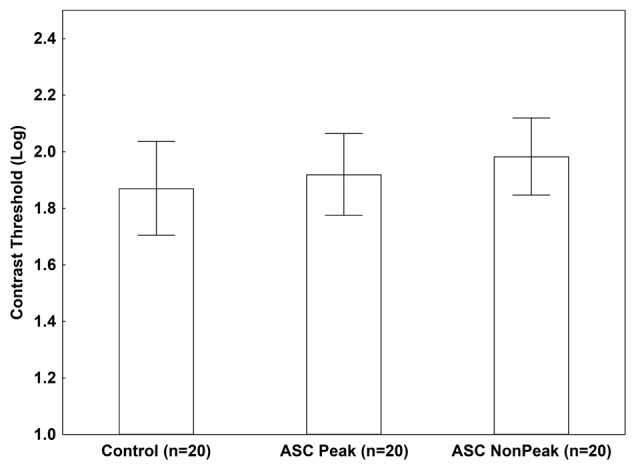
Mean log-contrast threshold (95% confidence intervals) in people with autism spectrum conditions (ASC) exhibiting a Block Design peak performance (ASC Peak), an average Block Design performance (ASC Non-Peak), and neurotypical (non-autistic) control volunteers. In this condition, a single Gabor stimulus was presented without masks. There were no statistically significant between-group differences (*p* > 0.5).

When an isolated Gabor patch was presented without masks, we found no significant difference among the groups (ASC Peak, ASC Non-Peak, and controls) in the contrast threshold (*p* = 0.54; Figure 2).

When the target Gabor patch was presented with masks in the perceptual task, there were significant main effects of group (*F*_(2,57)_ = 20.00, *p* < 0.001, *η*^2^ = 0.41) and target-mask distance (*F*_(2,114)_ = 567.56, *p* < 0.001, *η*^2^ = 0.91). The interaction between group and target-mask distance was also significant (*F*_(4,114)_ = 9.60, *p* < 0.001, *η*^2^ = 0.25).

**Figure 3:**
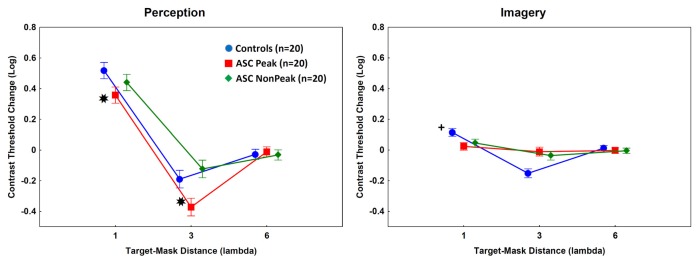
Mean log-contrast threshold changes (masks present minus isolated target stimuli). Error bars denote 95% confidence intervals. Negative values indicate facilitation by masks (lower contrast threshold for the target when the masks are presented relative to an isolated target). Perception: *Contrast threshold changes were significantly lower in the ASC Peak group (people with ASC exhibiting a Block Design peak performance) relative to the control individuals at 1λ and 3λ [Tukey’s Honestly Significant Difference (HSD) tests, *p*s < 0.01]. Imagery: ^+^The ASC Peak and ASC Non-Peak (people with autism spectrum disorders exhibiting an average Block Design performance) groups showed weaker inhibition at 1λ (lower contrast threshold changes) and weaker facilitation (higher contrast threshold changes) at 3λ relative to the controls (Tukey’s HSD tests, *p*s < 0.01).

As shown in Figure 3, the contrast threshold changes were significantly lower in the ASC Peak group relative to the control individuals at 1λ (less inhibition) and 3λ [more facilitation; Tukey’s HSD tests, *p*s < 0.01; *d* (1λ) = 1.5; *d* (3λ) = 1.6]. No such differences were observed when the ASC Non-Peak subjects were compared with the control group [*p*s > 0.3; *d* (1λ) = 0.5; *d* (3λ) = 0.7]. Finally, when the ASC Peak and ASC Non-Peak groups were directly compared, we found no differences at 1λ (*p* = 0.29; *d* = 0.6), whereas at 3λ the contrast threshold change was significantly lower in the ASC Peak group than in the ASC Non-Peak group (*p* < 0.01; *d* = 1.7; Figure 3).

**Figure 4:**
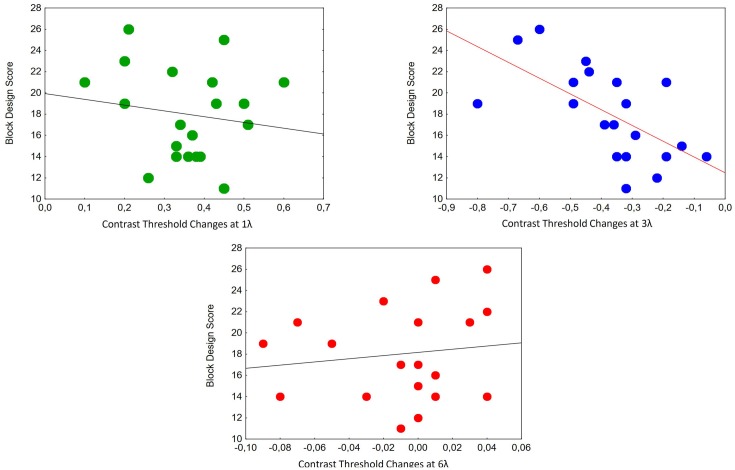
Correlations between contrast threshold changes and Block Design Scores at different target-mask distances in the perception task in individuals with ASC exhibiting a Block Design peak performance (*n* = 20). A significant relationship was found at 3λ (partial *r* = −0.65, *p* < 0.01).

We calculated the correlations between contrast threshold changes and Block Design Scores at different target-mask distances. A significant relationship was found at 3λ in the ASC Peak group (partial *r* = −0.65, *p* < 0.01; Figure 4).

We also investigated whether facilitation and inhibition were significant at the different target-mask distances by comparing contrast thresholds at each distance with the baseline (no mask) value. These analyses indicated significant inhibition at 1λ and facilitation at 3λ in each group (controls: 1λ: *t*_(19)_ = −23.13, *p* < 0.001, *d* = 1.4; 3λ: *t*_(19)_ = 13.97, *p* < 0.001, *d* = 0.7; ASC Peak: 1λ: *t*_(19)_ = −13.15, *p* < 0.001, *d* = 1.2; 3λ: *t*_(19)_ = 9.22, *p* < 0.001, *d* = 1.1; ASC Non-Peak: 1λ: *t*_(19)_ = −14.94, *p* < 0.001, *d* = 1.6; 3λ: *t*_(19)_ = 4.75, *p* < 0.01, *d* = 0.4). At 6λ, we found no significant facilitation in either group (*p*s > 0.1).

### Imagery

In the imagery task, the main effect of group was not significant (*p* = 0.56). However, the main effect of target-mask distance (*F*_(2,114)_ = 104.37, *p* < 0.001, *η*^2^ = 0.65) and the two-way interaction between group and target-mask distance (*F*_(4,114)_ = 33.52, *p* < 0.001, *η*^2^ = 0.54) were significant. The *post hoc* tests indicated that the ASC Peak and ASC Non-Peak groups displayed minimal inhibition at 1λ and weak facilitation at 3λ as compared with the controls [*p*s < 0.01; ASC Peak vs. controls: *d* (1λ) = 1.4; *d* (3λ) = 2.3; ASC Non Peak vs. controls: *d* (1λ) = 0.9; *d* (3λ) = 1.6]. There were no significant differences between individuals with ASC Peak and ASC Non-Peak [*p*s > 0.5; *d* (1λ) = 0.4; *d* (3λ) = 0.3; Figure 3].

When facilitation and inhibition were investigated at the different imagined target-mask distances by comparing contrast thresholds at each imagined distance with the baseline (no imagined mask) value, significant effects were found in the control group at 1λ (*t*_(19)_ = −7.57, *p* < 0.01, *d* = 0.5), at 3λ (*t*_(19)_ = 8.02, *p* < 0.01, *d* = 0.5), but not at 6λ (*p* > 0.1). In the ASC groups, there were no similar effects (*p*s > 0.1).

### Fatigue and Motivation

Individuals with ASC and healthy control subjects exhibited statistically similar scores on the MFI (before the experiment: ASC Peak: 37.4 (*SD* = 4.9), ASC Non-Peak: 38.3 (*SD* = 5.1), controls: 36.9 (*SD* = 4.6); after the experiment: ASC Peak: 36.3 (*SD* = 5.0), ASC Non-Peak: 38.0 (*SD* = 5.2), controls: 36.0 (*SD* = 4.7); ANOVA main effects of group and assessment time: *p*s > 0.5). The MFI scores did not correlate with perception, imagery, and neuropsychological performances (*p*s > 0.5).

## Discussion

The results from the control group are in keeping with the findings of prior experiments (Polat and Sagi, 1993). However, we only found partial support for our hypotheses in ASC. In accordance with the results of Kéïta et al. (2011), we observed higher facilitation of contrast detection in ASC relative to the controls when the target-mask distance was intermediate (3λ). However, this effect was confined to the ASC Peak group. In addition, Block Design performances specifically correlated with lateral facilitation in the ASC Peak group. This suggests that altered developmental trajectories, leading to outstanding higher-level visuoconstructional abilities, may also result in the enhancement (less inhibition) of lateral connections in the V1. From another point of view, atypical higher-level perception may stem from altered low-level processes in ASC (Perreault et al., 2015). Vandenbroucke et al. (2008) used an electrophysiological and neural network approach to map feedforward, horizontal, and recurrent feedback processing in ASC. They found abnormal object boundary detection as early as 120 ms after stimulus presentation, which may be a marker of dysfunctional lateral connections in early visual areas. Interestingly, ASC individuals were characterized by an enhanced subsequent occipital activity (225 ms), whereas recurrent feedback processing from extrastriate areas (260 ms) was spared (Vandenbroucke et al., 2008).

We also found weak inhibition at small target-mask distances (1λ) in the ASC Peak group. This is consistent with a previous functional magnetic resonance imaging study demonstrating a correlation between weaker surround suppression in the V1 and autistic traits in the general population (Flevaris and Murray, 2014), although brain imaging studies using the lateral masking paradigm have not been performed. Therefore, the interpretation of our behavioral results at the neuronal level is indirect. However, Dickinson et al. (2018) showed that greater severity of autistic symptoms was associated with increased short-range lateral inhibition. The authors concluded that lateral connections were not generally dysfunctional in ASC, and subtle alterations and individual variations might explain the heterogeneity of visual perceptual phenotype (Bertone et al., 2005; Dickinson et al., 2018). These divergent results may stem from different methods and from the heterogeneity of ASC.

Classic models suggest that the facilitatory effect of collinear masks is due to the spreading of excitatory signals from the mask-responsive to the target-responsive cells *via* lateral connections in the V1 (Livingstone and Hubel, 1984; Gilbert and Wiesel, 1989; Polat and Sagi, 1993). Feedback from higher cortical areas may strengthen the attentional modulation of collinear masks and targets (Gilbert et al., 2000; Freeman et al., 2003), although lateral facilitation seems to be too fast for such feedback effects (Yu et al., 2002). Specifically, lateral facilitation by collinear masks was demonstrated at a stimulus duration as brief as 8 ms, a timing too short for cortical feedback (Yu et al., 2002). According to the models of temporal dynamics, there are two possible components of facilitation. The first is a fast and short-acting component of facilitation that involves a single spatially-elongated perceptual channel defining the classic receptive field without long-range connections (Georgeson and Georgeson, 1987). The second is a delayed and long-acting component that is based on long-range connections between collinear flanker masks and target (Polat and Sagi, 2006). Huang and Hess (2008) showed that these models are not sufficient and that the dynamics of perceptual facilitation are both fast and sustained. The authors proposed a two-component model of facilitation consisting of a rapid signal across large retinal distances based on feedback from higher centers and a sustained, lower-level response involving the temporal integration of locally responsive mechanisms (Huang and Hess, 2008). Therefore, the main point of the Huang and Hess (2008) model, which is based on a series of psychophysical studies, is that facilitation is neither delayed and long-lasting, not fast and short-lived. Instead, facilitation is fast and sustained. Contrary to the classic view, the fast signal is based on feedback, whereas the sustained response is mediated by low-level mechanisms. In sharp contrast to lateral facilitation, masks can inhibit target detection if the target-mask distance is small (<2λ). This lateral inhibition is thought to be related to short-range inhibitory connections (Blakemore et al., 1970; Polat and Sagi, 1993; Shushruth et al., 2013). At the behavioral level, our results indicate that both excitatory and inhibitory lateral connections are altered in ASC Peak, characterized by enhanced excitation at intermediate target-mask distances (3λ) and reduced inhibition at small target-mask distances (1λ). This raises the possibility that autism-related developmental alterations in visual cortical circuits are not confined to a single mechanism, and that these can be detected especially in individuals with ASC who display exceptional visuoconstructional abilities.

We also identified a robust dissociation between perception and imagery: individuals with ASC, irrespective their visuoconstructional abilities, displayed a negligible imagery effect. It is possible that they failed to imagine the masks or the imagined masks failed to modulate the target. However, given that there were normal (ASC Non-Peak) or enhanced (ASC Peak) lateral interactions in the perceptual task, it is not likely that successfully imagined masks failed to act on the target. Overall, the dissociation between perception and imagery indicates enhanced early bottom-up perception and dampened top-down control in the ASC Peak group, whereas in the ASC Non-Peak group, weak top-down control was accompanied by unaltered perception.

Our results provide a plausible explanation for an important controversy in the literature. While Kéïta et al. (2011) reported increased lateral facilitation in individuals with ASC relative to controls, Jachim et al. (2015) found the opposite phenomenon, that is, weak facilitation in ASC. Notably, the sample of Jachim et al. (2015) included individuals with Asperger’s syndrome, which was not the case in the Kéïta et al.’s (2011) study. Differences in the experimental design could also contribute to the discrepancy of results. Kéïta et al. (2011) used long exposure time and added gray scale noise to the stimulus display, raising the possibility that noise differently interfered with perceptual processing in ASC and healthy controls. In our study, the stimulus exposure time was short, and no noise was applied, excluding the possibility that these parameters accounted for the difference in previous studies. Moreover, we controlled individual variations in cognitive developmental patterns (ASC Peak vs. ASC Non-Peak). However, it cannot be excluded that individuals with Asperger’s syndrome exhibit a distinct pattern of lateral masking performance. Furthermore, it is important to take into account that our participants were male, and the results may not be generalized to female individuals.

The finding that heightened Block Design performance was associated with poor imagery seems to be counterintuitive, because in a previous study Block Design performance was related to a more efficient imagery and manipulation of mental representations (Soulières et al., 2011). The authors conducted mental imagery experiments in 23 individuals with ASC (11 with Block Design peak) and 14 matched neurotypical controls. In the first experiment, participants were asked to imagine a letter inside a circle, whereas the second experiment included mental rotation tasks with two- and three-dimensional shapes, hands and letters. Individuals with ASC, especially those with Block Design peak, were more accurate in the imagery tasks relative to the controls. These results can be explained in the framework of a global advantage in perceptual processing (enhanced perceptual functioning model) or by an eminence in veridical mapping, which refers to the ability to detect isomorphisms among objects (Soulières et al., 2011). However, mental imagery abilities depended on the task type: the performance was more pronounced when the task included the mental rotation of three-dimensional objects rather than more simple two-dimensional shapes, hands, or letters (Soulières et al., 2011). Therefore, it is possible that imagery is even less effective in the case of low-level luminance contrast gratings, as shown in the present study. At the neuronal level, there is evidence that people with ASC show higher activation in a widespread parietal and occipital network during the Block Design task relative to non-autistic controls, but this enhanced neuronal network does not include the V1, which is critical in the lateral masking task (Polat and Sagi, 1993; Kana et al., 2013).

Decreased performances on the mental imagery task may be related to the impaired top-down control of information processing in ASC. At the neuronal level, it may be due to deficits in visual cortical areas, control areas (prefrontal cortex), or the connectivity between the sensory and control regions (Frith, 2003; Loth et al., 2010; Cook et al., 2012; Van Der Cruys et al., 2018). Our study could not explore these possibilities at the neural level, and it is not clear whether individuals with ASC failed to imagine the masks or the successfully imagined mask failed to exert an effect on the target. However, previous studies indicated that people with ASC exhibit normal (Kana et al., 2006) or even enhanced visual imagery (Soulières et al., 2011) using more complex stimuli. It has also been shown that non-visual cognitive control might play a compensatory role in imagery in ASC. When participants were asked to memorize a map of a fictitious island, verbal IQ and working memory were positively associated with image scanning performance in ASC, but not in healthy controls (Maras et al., 2014). In the present study, verbal compensation was not possible because of the simplicity of stimuli.

In the framework of the Bayesian decision theory, Pellicano and Burr (2012) suggested that atypical perceptual experiences in ASC can be explained by a deficient interaction between incoming sensory information and a prior knowledge of the world. Specifically, attenuated top-down influences (Bayesian “hypo-priors”) may induce a hyper-realistic perception, which is weakly influenced by past experiences. In our paradigm, prior experiences did not influence the detection of the target stimulus when participants were requested to retrieve and to imagine the previously presented masks. This suggests that the diminished effect of prior experiences can be revealed at the early stage of visual processing in ASC.

The general loss of imagery in ASC may stem from nonspecific factors (e.g., a misunderstanding of the instructions or a lack of motivation), and this is a main limitation of the study. However, individuals with ASC excellently performed on challenging neuropsychological and psychophysical tests in this study, even displaying better performances than healthy control subjects in some domains. Generally, as discussed above, individuals with ASC do not show impaired visual mental imagery (Kana et al., 2006; Soulières et al., 2011; Maras et al., 2014). The imagery condition was matched to the perception condition in task structure and general difficulty, and we made sure during the practice block that individuals with ASC understood the task correctly. Moreover, in our previous study, individuals with schizotypal personality disorder, who often find testing situations very challenging due to their decreased motivation, distractibility, and suspiciousness, exhibited enhanced (more effective) mental imagery relative to control participants, which is against the possibility that the imagery task is more difficult to understand or motivationally more demanding than the perceptual task (Maróthi and Kéri, 2018). Finally, a rating scale for fatigue and motivation did not indicate significant differences between individuals with ASC and healthy control participants.

In conclusion, our results provide psychophysical evidence for a unique pattern of lateral interactions in the V1 of individuals with ASC Peak: a decreased inhibition at short distance (1λ of target-mask distance) and an enhanced facilitation at intermediate distances (3λ of target-mask distance). The decreased horizontal inhibition in early-stage vision may be relevant to several assumptions of the enhanced perceptual theory and the weak central coherence hypothesis of autism (Happé and Frith, 2006; Mottron et al., 2006). Because of the decreased inhibition of adjacent stimuli (masks) on a central target, ASC Peak individuals may exhibit a detail-focused processing rather than a holistic perception of larger images (Happé and Frith, 2006). Specifically, decreased lateral inhibition exerted on a small spatial region (i.e., the target Gabor patch in the lateral masking task) may lead to the pop-out and enhanced perceptual awareness of this spot. From another point of view, low inhibition and high facilitation in the lateral connections of the V1 may lead to a better detection of elementary visual features and simple contours (Mottron et al., 2006; Loffler, 2008). The biological bases of these findings remain to be explored: inhibition-excitation ratio in the V1 is a possible target for future investigations (Robertson et al., 2016).

## Data Availability Statement

The datasets generated for this study are available on request to the corresponding author.

## Ethics Statement

The studies involving human participants were reviewed and approved by National Medical Research Council. The patients/participants provided their written informed consent to participate in this study.

## Author Contributions

SK, KC, and RM designed the study. RM coordinated data collection and measurements. RM and SK analyzed the data. RM, KC, and SK wrote the first draft of the article, which was reviewed, edited, and approved by both authors.

## Conflict of Interest

The authors declare that the research was conducted in the absence of any commercial or financial relationships that could be construed as a potential conflict of interest.
